# Suppression of hepatic stellate cell activation through downregulation of gremlin1 expression by the miR-23b/27b cluster

**DOI:** 10.18632/oncotarget.13365

**Published:** 2016-11-15

**Authors:** Xian-Yi Zeng, Yan-Qiong Zhang, Xiao-Min He, Lin-Yan Wan, Hu Wang, Yi-Ran Ni, Jie Wang, Jiang-Feng Wu, Chang-Bai Liu

**Affiliations:** ^1^ The Institute of Cell Therapy, China Three Gorges University, Yichang, 443000, China; ^2^ Medical College, China Three Gorges University, Yichang, 443002, China; ^3^ The First People's Hospital of Yichang, Hubei Yichang, 443000, China; ^4^ Hubei Key Laboratory of Tumor Microenvironment and Immunotherapy, China Three Gorges University, Yichang, 443002, China; ^5^ Institute of Liver Diseases, China Three Gorges University, Yichang, 443002, China

**Keywords:** hepatic fibrosis, TGF-β, BMP-7, miR-23b/27b cluster, siRNA

## Abstract

The imbalance between transforming growth factor β and bone morphogenetic protein 7 signaling pathways is a critical step in promoting hepatic stellate cell activation during hepatic fibrogenesis. Gremlin1 may impair the balance. Something remains unclear about the regulatory mechanisms of gremlin1 action on hepatic stellate cell activation and hepatic fibrosis. In the current study, gremlin1 overexpression promotes activation of hepatic stellate cells. Knockdown of gremlin1 with siRNAs suppresses hepatic stellate cell activation and attenuates hepatic fibrosis in rat model. Our results also show that miR-23b/27b cluster members bind to 3′-untranslated region of gremlin1 resulting in reduction of *transforming growth factor β, α-smooth muscle actin* and *collagenI α1/2* gene expression. Our findings suggest that gremlin1 promotes hepatic stellate cell activation and hepatic fibrogenesis through impairment of the balance between transforming growth factor β and bone morphogenetic protein 7 signaling pathways. The miR-23b/27b cluster suppresses activation of hepatic stellate cells through binding gremlin1 to rectify the imbalance.

## INTRODUCTION

Hepatic fibrosis is a common response to chronic liver injury, ultimately leading to cirrhosis and even hepatocellular carcinoma (HCC). Hepatic stellate cells (HSCs) are known to play a pivotal role in the fibrogenesis process. In chronic liver injury, HSCs are activated, followed by secretion of inflammatory cytokines and upregulation of extracellular matrix (ECM) synthesis, leading to significant ECM deposition in the liver. Transforming growth factor beta (TGF-β) is the key player in promoting activation of HSCs [1]. Bone morphogenetic proteins (BMPs), TGF-β superfamily members, exert antagonistic effects on the biological activities of TGF-β [2]. TGF-β and BMPs stimulate signal transduction pathways, leading to phosphorylation of Smad2/3 (TGF-β signaling pathway) or Smad1/5/8 (BMP signaling pathway), which, in combination with Smad4, shuttles into the nucleus to regulate downstream gene expression [3]. Overexpression of BMP-7 in rat models of renal failure reverses renal fibrosis by suppressing TGF-β-induced signaling [4]. TGF-β and BMP-7 therefore appear to exert opposite effects in hepatic fibrogenesis [5].

Gremlin is a target molecule of the TGF-β signaling pathway and is also antagonist to the BMP-7. It is a highly conserved protein with a similar three-dimensional structure to BMPs. Among three alternative splicing variants gremlin1, gremlin2 and gremlin3 [6], gremlin1 is the most common isoform. It is a 184 amino acid (a.a.) protein that exists as both secreted and cell-associated forms. The secreted form of gremlin1 is reported to be 20.7 kD and in the glycosylated state [7]. The protein has an eight-membered ring cysteine knot in which an additional cysteine residue near the knot forms the basis for homodimerization or heterodimerization with BMPs [8]. Gremlin1 binds directly to BMP-2/4/7 and prevents interactions with the respective receptors [9].

Gremlin1 was initially isolated from the neural crest of *Xenopus* and subsequently shown to be important in embryonic development [10]. Although gremlin1 expression usually decreases in adulthood, its levels are obviously increased in fibrosis diseases [11]. Data from earlier transcriptional profiling in a mouse hepatic fibrosis model support the utility of gremlin1 as a novel marker of liver fibrogenesis [12]. Gremlin1 and hairy enhancer of split-1 (Hes1) are elevated in human kidney epithelial cells stimulated by TGF-β and in diabetic nephropathy. In fact, the predicted microRNA (miRNA) binding elements and promoter structures of *gremlin1* and *Hes1* show significant similarities [13]. Previous research by our group showed that selective disruption of Hes1 suppresses the promoter activities of α-smooth muscle actin (α-SMA) and collagen Iα2 in activated HSCs [14]. These data imply that gremlin1 is able to promote TGF-β signal transduction, possibly leading to the imbalance between the TGF-β and BMP-7 signaling pathways in activated HSCs. During hepatic fibrogenesis, this may represent at least one possible reason for the sustained activation of HSCs even in conditions of lack of pathogenic insult.

Rat *gremlin1* mRNA (NM_019282.2) is 3809 nucleotides (nt) in length, including the 5′-untranslated region, coding sequence, and 3’-UTR (139, 555 and 3115 nt respectively). The relatively long 3’-UTR may provide a structural basis for binding of miRNAs. Endogenous miRNAs represent a broad class of 18–22 nt RNAs that inhibit the expression of target genes by negatively regulating the stability and translation of the corresponding mRNAs [15, 16]. It is reported that miR-133a suppresses Smad3/4-mediated TGF-β signaling [17]. But, the complex mechanisms underlying miRNA regulating of gremlin1 expression remain unknown. One member of the miR-23b/27b cluster, miR-27b, has the potential to inhibit fibrosis in pulmonary cells through targeting gremlin1 [18]. Being defined as a group of miRNA genes clustered together within a proximal distance on a chromosome indicated that its members may accomplish their functions synergistically throughout several biological processes [19]. The miR-23b/27b cluster, a prognostic marker in renal cell carcinoma [20], has shown to suppress the metastatic phenotype of castration-resistant prostate cancer cells [21]. Moreover, these microRNAs promote the growth of fetal hepatocytes via downregulation of Smads, and consequently TGF-β signaling [22]. However, it remains to be established whether the miR-23b/27b cluster has the capacity to inhibit gremlin1 expression via negative post-transcriptional regulation, and as a result, suppress HSC activation during hepatic fibrogenesis.

In this study, we hypothesized that gremlin1 stimulates HSC activation and is downregulated by the miR-23b/27b cluster, leading to alleviation of hepatic fibrosis via rectifying the imbalance between TGF-β and BMP-7 signaling. To examine this theory, we investigated the mechanisms underlying gremlin1 expression and HSC activation, specifically, the effects of gremlin1 downregulation on HSC activation and hepatic fibrosis *in vivo* and the capacity of the miR-23b/27b cluster to suppress gremlin1 expression. Our results collectively show that gremlin1 induces TGF-β expression and enhances TGF-β-mediated signaling and downstream gene expression. Notably, the miR-23b/27b cluster downregulates gremlin1 expression via binding to its 3′-UTR region, leading to suppression of HSC activation.

## RESULTS

### Gremlin1 modulates HSC activation

**Figure 1:**
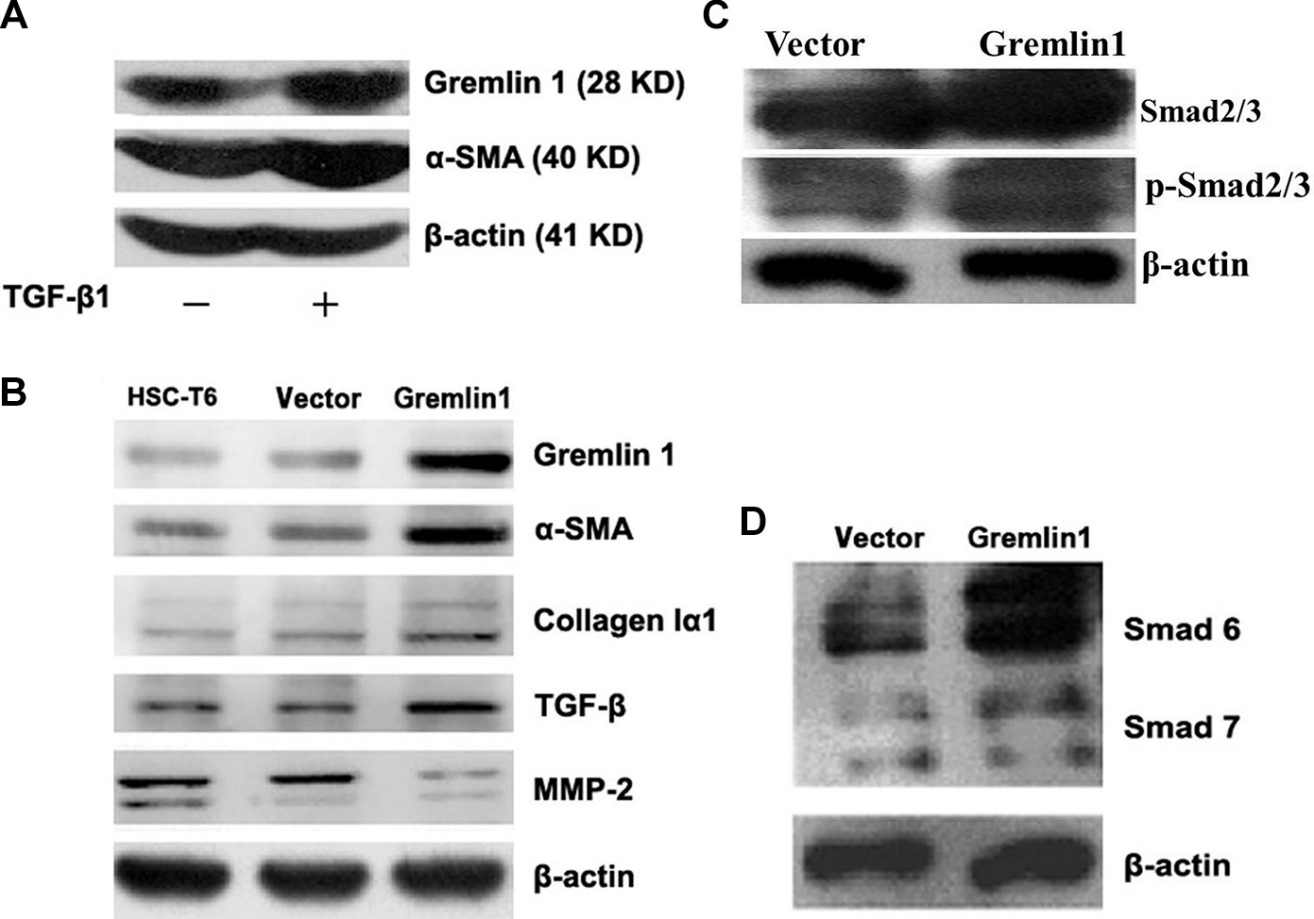
Overexpression of gremlin1 enhances HSC-T6 cell activation (**A**) Western blot shows increased gremlin1 and α-SMA expression in HSC-T6 cells stimulated with TGF-β1. (**B**) In gremlin1-HSC cells, expression of α-SMA, collagen Iα1 and TGF-β are increased, while MMP-2 is decreased. (**C**) Western blot shows upregulation and increased phosphorylation of Smad2/3 in Gremlin1-HSC cells. (**D**) Western blot shows that expression of Smad6, but not Smad7, is obviously increased in Gremlin1-HSC cells.

In view of the earlier finding from a transcriptome study that gremlin1 is evidently increased in activated HSCs from hepatic fibrosis model mice [12], we consistently observed increased expression of gremlin1 and α-SMA in HSC-T6 cells after TGF-β1 stimulation (Figure 1A). To determine the specific role of gremlin1 in activation of HSCs, both transient and stable expression of gremlin1 in HSC-T6 cells was achieved via transfection of pcDNA3.1-*gremlin1*, and western blot analysis was performed (Supplementary Figure 1A, 1B). The results revealed clear upregulation of α-SMA, a marker of activated HSCs (Figure 1B). Moreover, TGF-β expression was upregulated, and phosphorylation of Smad2/3 downstream of TGF-β-mediated signaling markedly increased. Additionally, not Smad7 of inhibitory Smad (I-Smad), but Smad6 expression was induced in HSC clones stably overexpressing gremlin1 (termed Gremlin1-HSC). Subsequently, collagen Iα1 was confirmed as being upregulated while matrix metalloproteinase 2 (MMP-2) was downregulated in Gremlin1-HSCs (Figure 1B, 1C, 1D). These data imply that gremlin1 overexpression promotes activation of HSCs, possibly via increased TGF-β and α-SMA expression, and enhanced TGF-β signal transduction and downregulation of MMP-2, leading to elevated ECM synthesis and decreased degradation.

**Figure 2:**
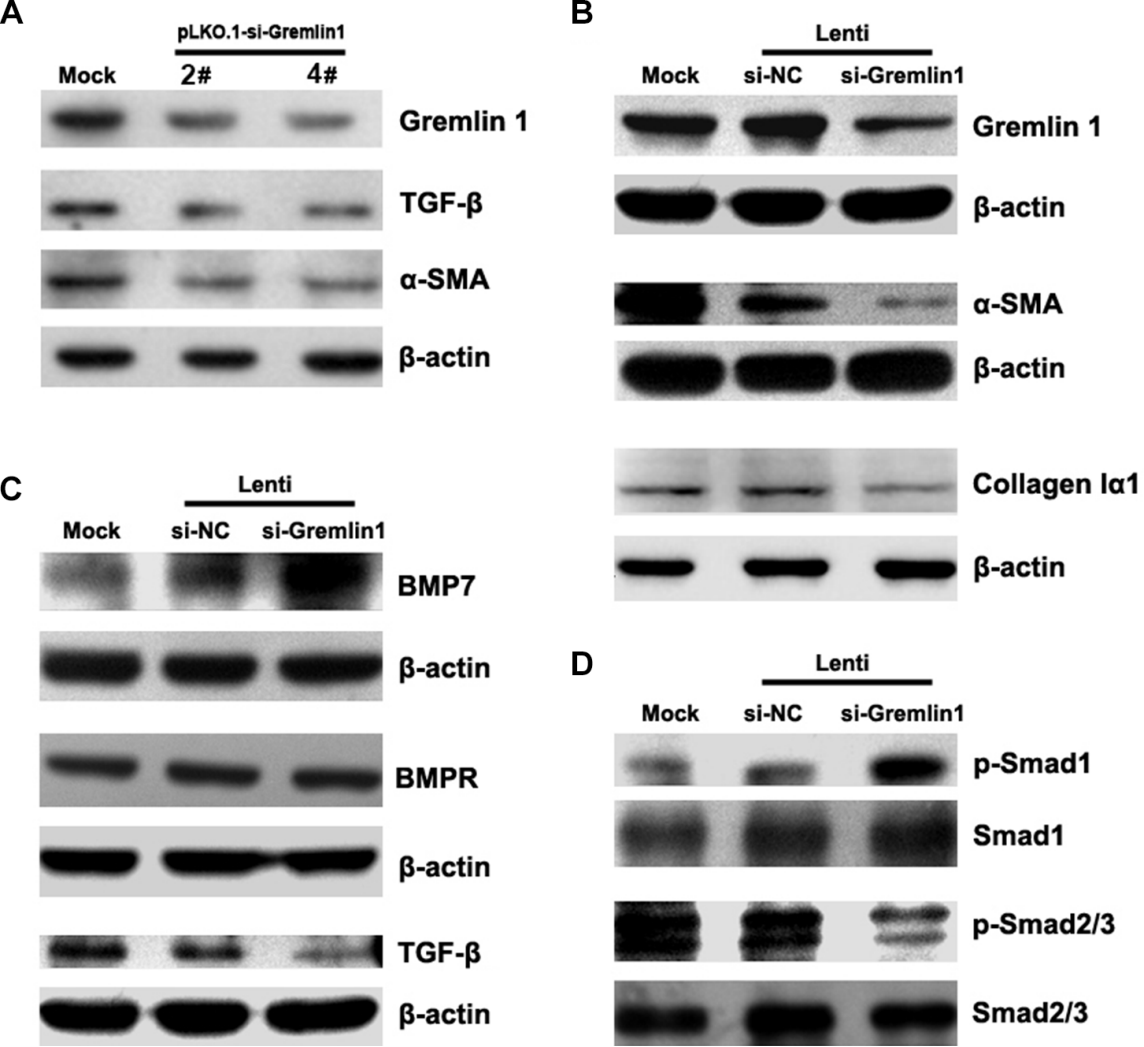
Knockdown of gremlin1 expression modulates BMP-7/TGF-β signaling leading to suppression of HSC activation (**A**) Transfection of HSC-T6 cells with gremlin1-specific shRNA-expressing vector, pLKO.1-si-Gremlin1-2# or 4#, led to partial downregulation of gremlin1 as well as TGF-β and α-SMA, observed via western blot. (**B**) Infection of HSC-T6 cells with gremlin1-specific shRNA lentivirus, Lenti-si-gremlin1 (1:1 mixture of pLKO.1-si-Gremlin1-2# and 4# packed virus particles) significantly suppressed gremlin1, α-SMA and collagen Iα1 expression. (**C**) Western blot shows that lenti-si-gremlin1 infection of HSC-T6 cells dramatically increases BMP-7, but not BMPR (BMP receptor) expression, and inhibits TGF-β expression, compared with Lenti-si-NC infection. (**D**) Western blots shows that phosphorylation of Smad1 is remarkably increased whereas that of Smad2/3 is significantly decreased in HSC-T6 cells infected with lenti-si-Gremlin1 virus.

To determine the effects of downregulation of gremlin1 on HSC activation, HSC-T6 cells were transiently transfected with *gremlin1*-specific shRNA expressing plasmids, and the siRNA efficiencies in *gremlin1* knockdown were assessed via semi-quantitative PCR and western blot (Supplementary Figure 1C, Table 1). The results showed that *gremlin1* siRNAs induced partial knockdown of *gremlin1* (Figure 2A; Supplementary Figure 1D, 1E). As predicted, TGF-β and α-SMA levels were significantly decreased in HSC-T6 cells with knockdown of *gremlin1* expression (Figure 2A; Supplementary Figure 1E). After infection with lenti-si-*gremlin1* virus, gremlin1 expression in HSC-T6 cells was significantly downregulated, as being determined with western blot (Figure 2B; Supplementary Figure 1F). To ascertain whether downregulation of gremlin1 leads to suppression of HSC activation, α-SMA and collagen Iα1 protein levels were detected. Expression levels of α-SMA and collagen Iα1 were remarkably decreased (*p* < 0.01) in the cells infected with lenti-si-Gremlin1 (Figure 2B; Supplementary Figure 1F). The results suggest that knockdown of *gremlin1* suppresses HSC activation, although the underlying pathogenic mechanisms remain to be clarified.

**Table 1:** Sequences of the scramble and shRNA candidates for gremlin1 specific siRNA

name	sequnce
Gremlin1-scrable, Top	5′-CCGGTCCCCAGGTACCGATCACAATCTCGAGATTGTGATCGGTACCTGGGGC TTTTTG-3′
Gremlin1-scrable, Bottom	5′-AATTCAAAAAGCCCCAGGTACCGATCACAATCTCGAGATTGTGATCGGTACCTGGGGA-3′
Gremlin1-siRNA1, Top	5′-CCGGATACCTGAAGCGAGATTGGTCTCGAGACCAATCTCGCTTCAGGTATTTTTTG-3′
Gremlin1-siRNA1, Bottom	5′-AATTCAAAAAATACCTGAAGCGAGATTGGTCTCGAGACCAATCTCGCTTCAGGTAT-3′
Gremlin1-siRNA2, Top	5′-CCGGGCCCTACTGCCAGCAGCTGATCTCGAGATCAGCTGCTGGCAGTAGGGCTTTTTG-3′
Gremlin1-siRNA2, Bottom	5′-AATTCAAAAAGCCCTACTGCCAGCAGCTGATCTCGAGATCAGCTGCTGGCAGTAGGGC-3′
Gremlin1-siRNA3, Top	5′-CCGGGCAGTGTCGTTGCATATCCTCTCGAGAGGATATGCAACGACACTGCTTTTTG-3′
Gremlin1-siRNA3, Bottom	5′-AATTCAAAAAGCAGTGTCGTTGCATATCCTCTCGAGAGGATATGCAACGACACTGC-3′
Gremlin1-siRNA4, Top	5′-CCGGGCACTATCATCAATCGCTTCTCTCGAGAGAAGCGATTGATGATAGTGCTTTTTG-3′
Gremlin1-siRNA4, Bottom	5′-AATTCAAAAAGCACTATCATCAATCGCTTCTCTCGAGAGAAGCGATTGATGATAGTGC-3′

### Downregulation of gremlin1 expression balances BMP-7 and TGF-β signaling

As gremlin1 is an antagonist of BMPs, a key question is whether its downregulation affects BMP-7 signaling in HSCs. Smad1, a mediator of BMP signaling, is phosphorylated upon BMP-stimulated signal transduction. BMP-7 expression was markedly upregulated and phosphorylation of Smad1 significantly increased in HSC-T6 cells infected with lenti-si-Gremlin1 virus on a western blot (*p* < 0.01) (Figure 2C, 2D; Supplementary Figure 1G, 1H). However, BMP receptor expression was not affected after knockdown of gremlin1 (*p* > 0.05) (Figure 2C; Supplementary Figure 1G). The results indicate that downregulation of gremlin1 expression increases the level of BMP-7 and enhances the associated signal transduction in HSCs.

As shown above, during hepatic fibrogenesis, gremlin1 is upregulated along with TGF-β expression and phosphorylation of Smad2/3, the major downstream signaling molecules of TGF-β. Western blot was conducted to evaluate whether downregulation of gremlin1 affects TGF-β-induced signaling indicated a significant decrease in TGF-β1 and Smad2/3 phosphorylation (*p* < 0.01) in HSC-T6 cells infected with lenti-si-Gremlin1 virus (Figure 2C, 2D; Supplementary Figure 1G, 1H). The findings suggest that although gremlin1 is a target of TGF-β signaling, knockdown of its expression can lead to downregulation of TGF-β. Further studies are required to elucidate the regulatory loop between gremlin1 and TGF-β.

### Downregulation of gremlin1 attenuates hepatic fibrosis in a SD rat model

**Figure 3:**
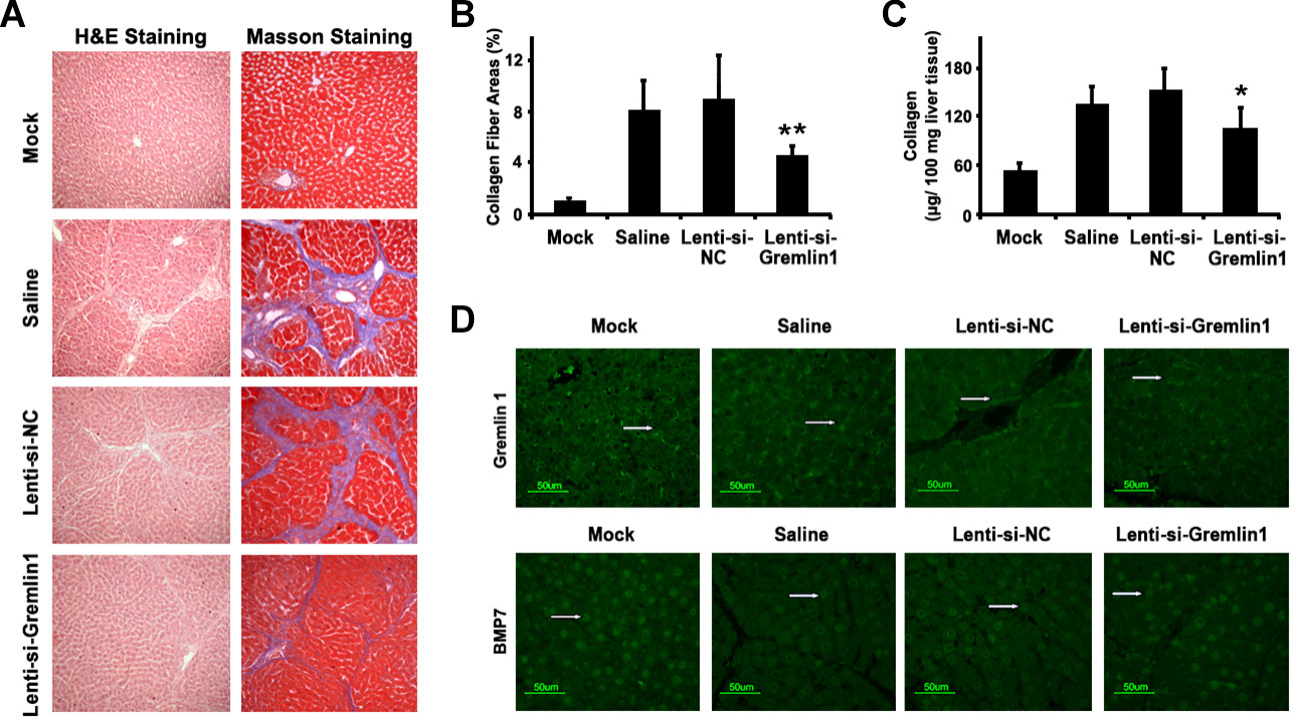
siRNA-mediated downregulation of gremlin1 expression alleviates hepatic fibrosis in a SD rat model (**A**) Histopathological changes in liver tissue from a CCl_4_-induced hepatic fibrosis SD rat model analyzed with H&E staining and deposition of collagens visualized with Masson staining. Compared with corn-oil or Lenti-si-NC virus infection, histopathological changes and collagen deposition were almost alleviated in model SD rats. (**B**) Quantification of the collagen fiber area revealed a significant decrease in lenti-si-Gremlin1-infected SD rats, compared with that of corn-oil-treated or lenti-si-NC-infected model SD rats. (**C**) Determination of collagen content with the QuickZyme total collagen kit revealed a significant decrease following lenti-si-Gremlin1 infection, compared to corn-oil-treated or Lenti-si-NC-infected model SD rats. (**D**) Immunofluorescence analysis revealed increased Gremlin1 expression (green fluorescence indicated by the white arrowhead) in liver tissue in hepatic fibrosis model SD rats or those treated with corn-oil or Lenti-si-NC virus infection, and significant downregulation in model SD rats with Lenti-si-Gremlin1 virus infection. BMP-7 expression (green fluorescence indicated by the white arrowhead) in liver tissue from hepatic fibrosis model SD rats or those treated with corn-oil or Lenti-si-NC virus infection was remarkably decreased, and restored in liver tissue from model SD rats infected with Lenti-si-Gremlin1 virus (*n* = 6 per group, model SD rats infected with Lenti-si-Gremlin1 versus Lenti-si-NC, *p < 0.05; **p < 0.01).

To investigate the effects of gremlin1 knockdown using specific siRNA on hepatic fibrosis *in vivo*, carbon tetrachloride (CCl_4_)-induced hepatic fibrosis model SD rats were established. Immunofluorescence analysis disclosed that in hepatic fibrosis model SD rat liver tissue, gremlin1 expression was remarkably elevated, compared with that in mock SD rats. Following lenti-si-Gremlin1 virus infection, gremlin1 expression in liver tissues of model SD rats was markedly suppressed (Figure 3D).

To explore whether downregulation of gremlin1 expression relieves hepatic fibrosis in hepatic fibrosis model SD rats, histological changes and collagen deposition in liver were studied. Infection of model SD rats with lenti-si-Gremlin1 virus resulted in marked attenuation of collagen synthesis and deposition in the liver (*p* < 0.05), compared to model SD rats injected with corn-oil or Lenti-si-NC (Figure 3A, 3B, 3C). To further verify whether the reversal of hepatic fibrosis of CCl4-induced model SD rats through siRNA knockdown of gremlin1 is associated with BMP-7, the expression of the protein in liver tissue was detected via immunofluorescence. Notably, the BMP-7 expression was decreased in model SD rats and those with Lenti-si-NC virus infection, compared to normal SD rats, but restored in model SD rats infected with lenti-si-Gremlin1 virus (Figure 3D). These data suggest that downregulation of gremlin1 expression not only suppresses HSC activation but also attenuates hepatic fibrosis via restoring the balance between BMP-7 and TGF-β signaling in CCl4-induced hepatic fibrosis model SD rats.

### The miR-23b/27b cluster downregulates gremlin1 expression in HSCs

**Figure 4:**
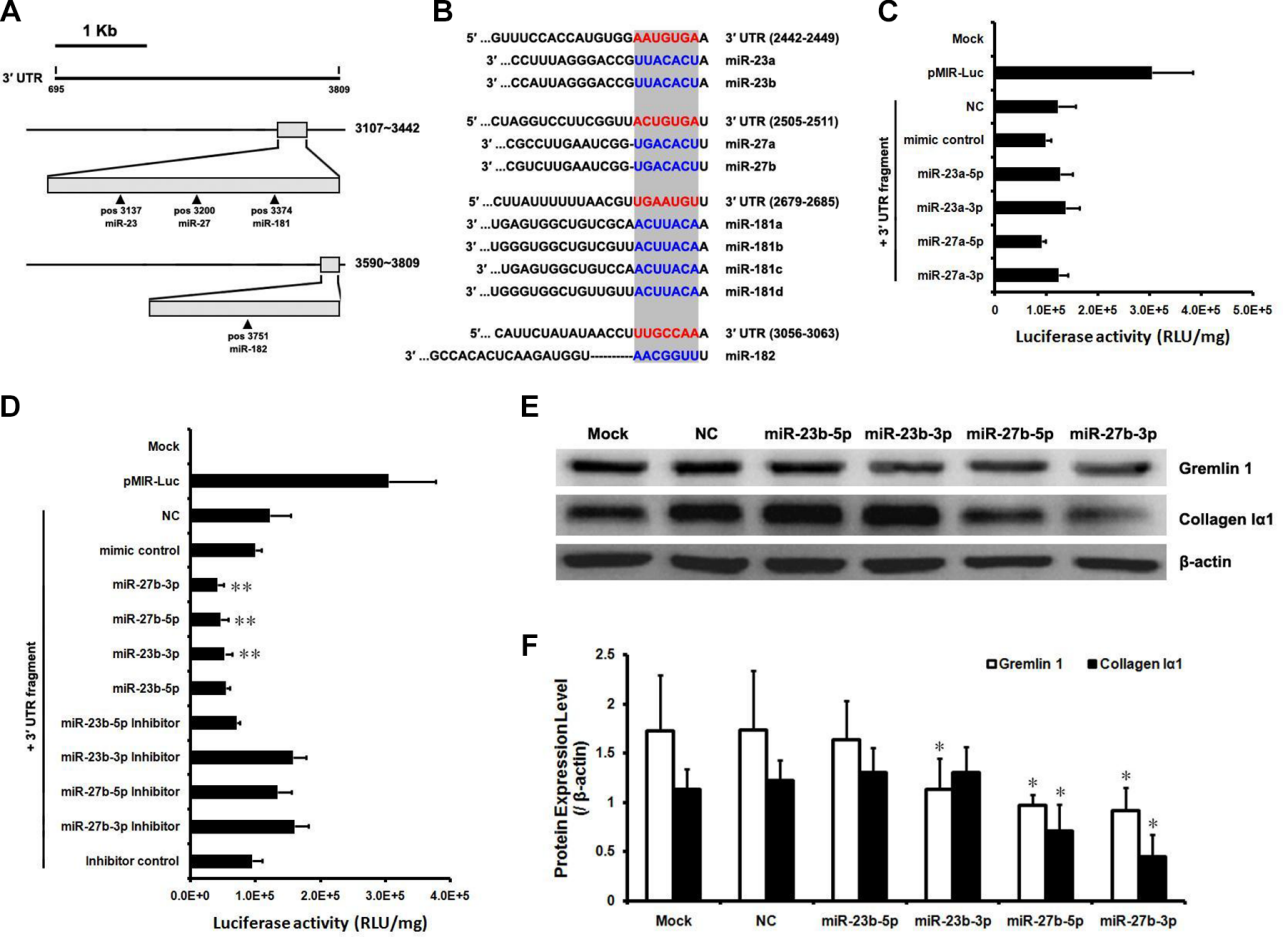
The miR-23b/27b cluster downregulates gremlin1 expression (**A**) Prediction of conserved miRNAs targeting the 3′-UTR of rat gremlin1 mRNA using Targetscan online service. Conserved binding sites for miR-23a/b, miR-27a/b and miR-181a/b/c/d were predicted in 3107–3442 nt region (G3-23-1) and for miR-182 in the 3590–3809 nt region (G3-23-3). (**B**) Nine conserved miRNAs, including miR-23a/b, miR-27a/b, miR-181a/b/c/d and miRNA-182, could recognize and bind the 3′-UTR of gremlin1 mRNA. (**C**–**D**) Verification of interactions between rat miR-23a/27a or miR-23b/27b and 3′-UTR of rat gremlin1 mRNA via determination of luciferase activities of HSC-T6 cells co-transfected with pMIR-Luc-3′-UTR and miRNA mimics or inhibitors. **p < 0.01 for comparing between three groups data: miRNA mimic comparing with mimic control; miRNA mimic comparing with its inhibitor; miRNA mimic inhibitor comparing with inhibitor control. (**E**–**F**) Western blot assay confirmed that gremlin1 is a cognate target of post-transcriptional repression by miR-23b/27b. Downregulation of gremlin1 expression led to inhibition of collagen Iα1 synthesis. Data in histogram figures represent means ± s.d (*n* = 3; *P < 0.05).

Gremlin1 has been shown to play an important pathophysiological role in liver fibrogenesis. Moreover, *gremlin1* mRNA has a relatively long 3’-UTR, providing a good structural basis for the binding of miRNAs that suppresses its expression. To predict whether rat miRNA candidates recognize and bind the target sequences in rat *gremlin1* mRNA 3′-UTR, Targetscan online service (http://www.targetscan.org/) was used to analyze conserved miRNAs and their target sequences. The context+ scores and features of the conserved miRNAs were further evaluated via Targetscan online prediction. In order, the miRNAs potentially contributing to downregulation of gremlin1 expression were identified as miR-27a/b, miR-23a/b, miR-181a/b/c/d and miR-182 (Figure 4A, 4B). Accordingly, the two clusters of miR-23a/27a and miR-23b/27b were selected for subsequent experiments.

HSC-T6 cells were co-transfected with a series of miRNA mimics (Table 2) and pMIR-Luc-3′-UTR to confirm whether the two miRNA clusters effectively bind the 3′-UTR region to downregulate gremlin1 expression. The results disclosed no obvious differences in luciferase activities between the cells treated with miR-23a-5p mimic (*p* > 0.05), miR-27a-5p mimic (*p* > 0.05) or miR-27a-3p mimic (*p* > 0.05), and those treated with negative control mimics. However, luciferase activity was significantly increased (*p* < 0.05) in the cells treated with the miR-23a-3p mimic (Figure 4C). Treatment with miR-23a/27a mimics did not decrease the luciferase activity of the cells transfected with pMIR-Luc-3’-UTR, suggesting that the miR-23a/27a cluster is not involved in downregulation of gremlin1.

**Table 2:** miRNA mimics and their inhibitors information (rat)

Name of miRNAs	Serial number of miRBase	Sequence of mature miRNAs and inhibitors
rno-miR-23b-5p	MIMAT0017099	Mimic: GGGUUCCUGGCAUGCUGAUUU Inhibitor: CCCAAGGACCGUACGACUAAA
rno-miR-23b-3p	MIMAT0000793	Mimic: AUCACAUUGCCAGGGAUUACC Inhibitor: UAGUGUAACGGUCCCUAAUGG
rno-miR-27b-5p	MIMAT0017101	Mimic: AGAGCUUAGCUGAUUGGUGAACAG Inhibitor: UCUCGAAUCGACUAACCACUUGUC
rno-miR-27b-3p	MIMAT0000798	Mimic: UUCACAGUGGCUAAGUUCUGC Inhibitor: AAGUGUCACCGAUUCAAGACG
Negative control		micrONTM miRNA mimic Ncontrol #22_Standard
Inhibitor Ncontrol		micrOFFTM miRNA inhibitor Ncontrol #22_Standard
Fluorescent labeling		micrONTM mimics Negative Control #22(5Cy3)

**Figure 5:**
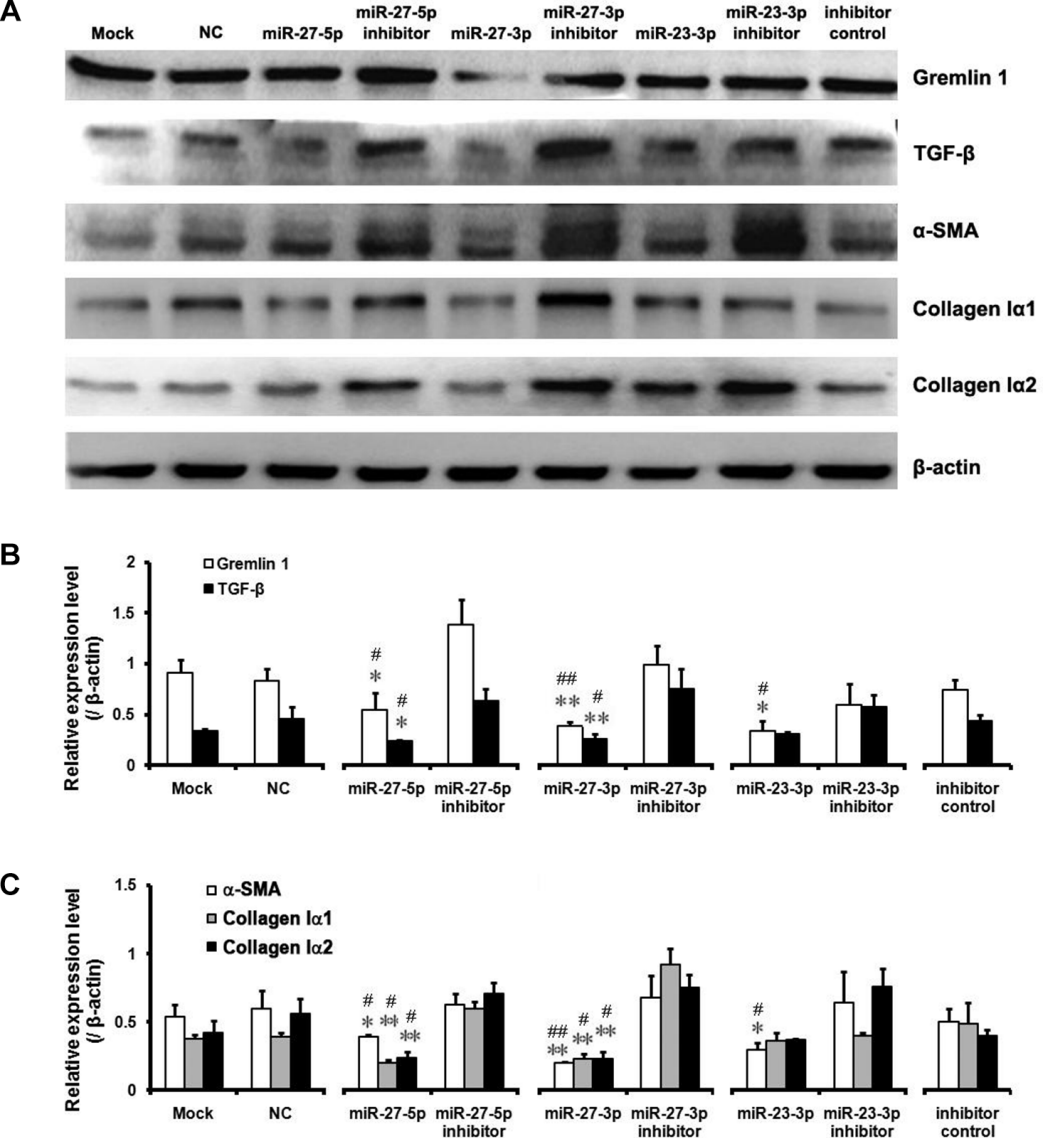
Downregulation of gremlin1 expression by the miR-23b/27b cluster leads to decreased expression of hepatic fibrosis-related factors (**A**–**C**) Expression levels of hepatic fibrosis-related factors, including α-SMA, TGF-β, collagen Iα1 and collagen Iα2, were critically assessed via western blot of the proteins from HSC-T6 cells transfected with miR-23b/27b mimics, inhibitors, negative control or inhibitor control, respectively. Relative protein level of Gremlin1, TGF-β, collagen Iα1, collagen Iα2 and α-SMA vs their inhibitors, respectively and all results were expressed as means ± SD (*n* = 3; *P < 0.05, **P < 0.01). Relative protein level of Gremlin1, TGF-β, collagen Iα1, collagen Iα2 and α-SMA vs their NC (Negative control), respectively and all results were expressed as means ± SD (*n* = 3; ^#^*P* < 0.05, ^##^*P* < 0.01).

Next, we analyzed the contribution of the miR-23b/27b cluster to suppression of gremlin1 expression. The results showed a significant decrease in luciferase activity of cells transfected with pMIR-Luc-3′-UTR followed by treatment with the miR-23b-3p (*p* < 0.01), miR-27b-5p (*p* < 0.01) and miR-27b-3p (*p* < 0.01) mimics, compared to cells treated with the negative control mimic or their inhibitors. Luciferase activity of cells transfected with pMIR-Luc-3′-UTR followed by treatment with the miR-23b-5p mimic was significantly decreased (*p* < 0.01), compared to those treated with the negative control mimics, but no significant increase (*p* > 0.05) was evident after treatment with its inhibitors, with no obvious changes (*p* > 0.05) between inhibitor and inhibitor control treatments (Figure 4D). These data suggest that miR-23b-3p, miR-27b-5p and miR-27b-3p bind to target sequences in the 3′-UTR and downregulate gremlin1 expression. The contribution of the miR-23b/27b cluster in downregulation of gremlin1 expression was further confirmed via western blot. Endogenous gremlin1 expression in HSC-T6 cells was downregulated in the presence of miR-23b-3p, miR-27b-5p and miR-27b-3p mimics (*p* < 0.05), but not the miR-23b-5p mimic (*p* > 0.05) (Figure 4E, 4F). To further verify the negative effects of the three miRNA mimics on gremlin1 expression, HSC-T6 cells were transfected with the three miRNAs mimic, their inhibitors, the negative control and inhibitor control, respectively. Western blot data confirmed that the three miRNA mimics downregulate gremlin1 expression (*p* < 0.05), compared with the negative control and inhibitors (Figure 5A, 5B).

### The miR-23b/27b cluster suppresses HSC activation through downregulating gremlin1 expression

As the miR-23b/27b cluster appears to directly inhibit gremlin1 expression, we rigorously analyzed its effects on HSC activation. Firstly, treatment with the miR-23b-3p, miR-27b-5p or miR-27b-3p mimics, compared to the corresponding inhibitors, led to downregulation of α-SMA in HSC-T6 cells (*p* < 0.05) (Figure 5A, 5C). As mentioned previously, TGF-β has a crucial role in activation of HSCs as well as expression of α-SMA. Accordingly, we assessed TGF-β expression after miRNA mimic treatment. Notably, treatment with both miR-27b-5p and miR-27b-3p mimics suppressed the expression of TGF-β on a western blot (*p* < 0.05) (Figure 5A, 5B).

Increased synthesis of collagens, the main components of ECM, is the most important pathological feature of activated HSCs in hepatic fibrosis. Western blot analysis of collagen Iα1 and Iα2 revealed a significant decrease in collagen Iα1 and Iα2 in HSC-T6 cells treated with the miR-27b-5p (*p* < 0.05) or miR-27b-3p (*p* < 0.01) mimic, but not those treated with the miR-23b-3p mimic (*p* > 0.05) (Figure 5A, 5C). These findings clearly suggest that the miR-23b/27b cluster suppresses activation of HSCs and inhibits collagen synthesis. However, further investigation is warranted to clarify the complex mechanisms underlying involvement of the miRNA 23b/27b cluster in suppression of gremlin1 expression and consequent regulation of HSC activation.

## DISCUSSION

Gremlin1 expression is obviously elevated in fibrosis diseases, such as interstitial fibrosis [23] idiopathic pulmonary fibrosis [11]. Recent studies have reported higher gremlin1 expression in cirrhosis than that in liver diseases [24] and a significant increase in gremlin1 levels in rat or mouse hepatic fibrosis models [12, 25]. An earlier transcriptome study by Boers *et al*. [12] revealed a marked increase in gremlin1 mRNA in activated HSCs from a hepatic fibrosis mouse model. In the current study, gremlin1 expression was enhanced in activated HSCs and in hepatic tissue of rat hepatic fibrosis model.

Gremlin1 overexpression in renal tubules enhances sensitivity of fibrosis in animal models [26]. Moreover, gremlin1 induces the expression of fibrosis-related proteins, such as α-SMA, collagen I and Smad2/3, in human lens epithelial cells [27]. It also triggers sustained Smad activation in tubular epithelial cells [28]. Silencing of gremlin1 relieves pancreatic fibrosis in chronic pancreatitis [29] and ameliorates renal damage in animal models [26]. Data from the current study have confirmed that overexpression of gremlin1 in HSCs significantly enhances TGF-β, α-SMA and collagen expression and suppresses MMP-2, the enzyme playing a critical role in degradation of ECM. Knockdown of gremlin1 expression in HSCs inhibits TGF-β, α-SMA and collagen I expression. It also reduces hepatic fibrosis in the CCl_4_-induced rat model. Accordingly, we conclude that gremlin1 expression induces HSC activation and increases collagen synthesis.Conversely, its knockdown inhibits HSC activation, leading to alleviation of hepatic fibrosis in a rat hepatic fibrosis model.

Upregulation of gremlin1 expression induces lung fibrosis in the mouse model, possibly via inhibition of BMP signaling and enhancement of TGF-β signal transduction [30]. Consistent with earlier findings, TGF-β induced gremlin1 expression in HSCs in our experiments. Overexpression of gremlin1 in HSCs not only promoted phosphorylation of Smad2/3, but also upregulated the TGF-β and the target molecule Smad6. On the other hand, silencing gremlin1 led to downregulation of TGF-β expression and inhibition of Smad2/3 phosphorylation in HSCs. Rodrigues-Diez *et al*. [28] reported that gremlin1 activated the Smad pathway, directly inducing TGF-β and contributing to renal fibrosis. It is speculated that as a TGF-β signaling target, gremlin1 overexpression in HSCs induces upregulation of TGF-β by activating Smad2/3 to form a positive regulation loop. The loop leads to sustained activation of the TGF-β/Smad2/3 signaling pathway. Our data further confirm that knockdown of *gremlin1*, both *in vitro* and *in vivo*, enhances the BMP-7 protein level and Smad1 phosphorylation. Other recent studies have indicated that gremlin1 inhibits BMP signaling, through not only direct interactions with BMPs resulting in blockage of ligand-receptor interactions [6], but also intracellular interactions with BMP precursor proteins to inhibit its maturation and biological activities [31]. In view of the findings, it is suggested that decreased levels of gremlin1 protein lead to the release of more frees BMP-7. Following activation of BMP-7/Smad1/5/8 signaling, transcription of target genes, such as *Smad6* and *Smad7*, is enhanced [32]. BMP7 signaling is regulated by I-Smads (including Smad6 and Smad7). In fact, I-Smads inhibit BMP rather than TGF-β signaling [33]. High levels of gremlin1 may block BMP-7 activity through upregulation of Smad6 expression, since its overexpression in HSCs promoted Smad6, but not Smad7 expression in the current study. Human proximal tubule epithelial cells with knockdown of *gremlin1* are able to recover BMP-7 signaling activity [34]. Moreover, BMP-7 signaling decreases the expression of α-SMA and collagen Iα2, and suppresses nuclear accumulation of the TGF-β signaling mediator, Smad3 [5]. Exogenous BMP-7 exerts an anti-fibrogenic effect in hepatic fibrosis model rats [35]. Thus it suggest that during hepatic fibrogenesis, high concentrations of TGF-β in inflammation responses upregulate gremlin1 expression and receive positive feedback from gremlin1, leading to TGF-β expression. Simultaneously, upregulation of gremlin1 inhibits BMP-7 that antagonizes TGF-β signaling, further intensifying TGF-β signaling activity, in turn, accelerating hepatic fibrogenesis.

Since siRNA-mediated silencing of *gremlin1* inhibits activation of HSCs and relieves hepatic fibrosis *in vivo*, triggering the endogenous regulatory mechanism to silence *gremlin1* may be more in line with clinical needs. For this purpose, we identified miR-27b and miR-23b were evidently involved in downregulation of gremlin1 expression in HSCs. Binding of miR-27b and miR-23b to gremlin1 mRNA 3′-UTR led to a marked decrease in gremlin1 expression as well as inhibition of α-SMA, collagen Iα1, Iα2 and TGF-β expression in HSCs. Recent studies have demonstrated that the miR-23b/27b cluster directly downregulates epidermal growth factor receptor and hepatocyte growth factor receptor [36]. It alsoinhibits TGF-β signaling pathway and promotes the BMP-7 signaling pathway through targeting Smad3 and Smad4 [22]. Accordingly, we conclude that the miR-23b/27b cluster inhibits HSC activation by directly binding gremlin1 mRNA and downregulating its expression. It may also negatively regulate Smad3 and Smad4 expression to suppress HSC activation.

**Figure 6:**
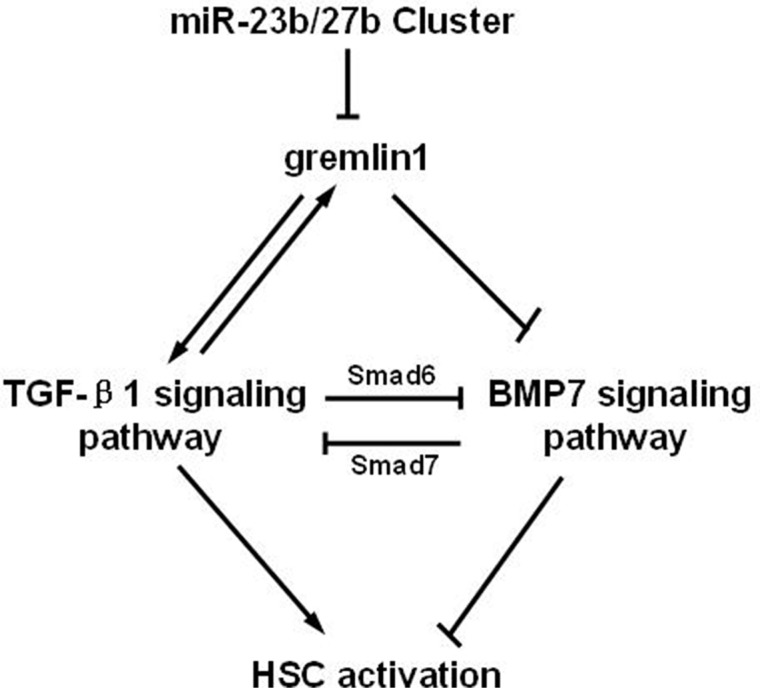
Schematic representation of suppression of HSC activation through downregulation of gremlin1 expression by the miR-23b/27b

In summary, our findings clearly demonstrate that gremlin1 promotes HSC activation and hepatic fibrosis through impairing the balance between BMP-7 and TGF-β1 signaling. The miR-23b/27b cluster suppresses HSC activation by directly binding gremlin1 mRNA 3′-UTR and downregulates gremlin1 expression to correct the imbalance of TGF-β and BMP-7 signal transduction (Figure 6). Data from the current study highlight the utility of the miR-23b/27b cluster as a novel target for manipulation in clinical therapy of hepatic fibrosis.

## MATERIALS AND METHODS

### Preparations for recombinant plasmids, lentivirus particles and mimic miRNAs

The pcDNA3.1-*gremlin1* was constructed by inserting CDS of rattus norvegicus *gremlin1* (NM_019282.3) into MCS of pcDNA 3.1 (invitrogen, Thermo Scientific, USA) between restriction enzyme sites *Kpn I* and *Xho I*. Luciferase reporter gene expression plasmid pMIR-*Luc-3*′*-UTR* was constructed by inserting full length 3′-UTR of rat *gremlin1* mRNA into the MCS of pMIR-PEPORT^TM^ Luciferase (pMIR-Luc) plasmid between *Hind III* and *Sac I* at the downstream of Luciferase open reading frame.

The pLKO.1-si-*gremlin1* 1#, pLKO.1-si-*gremlin1* 2#, pLKO.1-si-*gremlin1* 3#, pLKO.1-si-*gremlin1* 4# and pLKO.1-si-NC were constructed by inserting the cDNA encoding, the candidates of gremlin1 specific shRNA and scramble shRNA into cloning site between *Age I* and *EcoR I* in PLKO.1 vector respectively. The cDNAs encoding shRNAs (1#, 2#, 3#, 4# and the scrambled shRNA) are annealed at two complementary DNA strands designed in Ambion online service (listed in the Table 1) (Ambion, Thermo Scientific, USA).

All the DNA segments including the primers for cDNAs cloning and RT-PCR used in this study were synthesized by Sangon Biotech (Shanghai, China), and all the recombinant plasmids were further confirmed by DNA sequencing (Sangon Biotech, Shanghai, China)

After PLKO.1-si-*gremlin1*: 2# and 4# were identified as effective siRNA specific to *gremlin1*, the 2#, 4# and scrambled shRNA (PLKO.1-si-NC) were chosen to pack lentivirus particles of lenti-si-*gremlin1* 2#, lenti-si-*gremlin1* 4# and lenti-si-NC by GenePharma (Shanghai, China). 10^9^ TU/ml of each lentivirus particles were stored at −80°C for future use.

The mimic sequences of miR-23a/27a, miR-23b/27b and inhibitors of miR-23b/27b were got from the miRBase (http://www.mirbase.org/) online server prediction. A series of mimic miRNAs informations are shown in the Table 2. These mimic miRNAs, segments of inhibitor and fluorescent labeling mimic miRNA were synthesized by RiboBio Co. (Guangzhou, China).

### Cell culture, plasmid transfection and lentivirus infection of HSC-T6 cells

The HSC-T6 cell line was maintained in our laboratory. HSC-T6 cells were cultured in Dulbecco's Modified Eagle's Medium (DMEM) supplemented with 10% newborn calf serum (NBCS) (Invitrogen, Thermo Scientific, USA). Cells were seeded in a six-well plate (for western blot) or 24-well plate (for PCR or luciferase assays) (Greiner, Frickenhausen, Germany) at a density of 5 × 10^5^ cells per well in DMEM in a humidified atmosphere containing 5% CO_2_ for 24 h at 37°C.

HSC-T6 cells were transiently transfected with pcDNA3.1-*gremlin1*, or the *gremlin1*-specific short hairpin RNA (shRNA) expression plasmid based on pLKO.1, or *gremlin1* 3′-UTR-regulated pMIR-Luc (pMIR-REPORTTM Luciferase) plasmid, respectively, using TurboFect Transfection Reagent (Thermo Scientific, USA), according to the manufacturer's instructions. The cells were collected at 24, 36, or 48 h post-transfection to perform reverse transcription PCR, luciferase activity and western blot analysis.

After screening with transient transfection, efficient shRNA expression constructs of pLKO.1-si-*gremlin1*-2#, 4# and scrambled short RNA (si-NC) were chosen to be packed into lentivirus particles (lenti-si-*gremlin1* 2#, lenti-si-*gremlin1* 4# and lenti-si-NC) (GenePharma Co, Shanghai, China).

In lentivirus infection experiments, HSC-T6 cells were infected with the lentivirus particle mixture from lenti-si-*gremlin1* 2# and lenti-si-*gremlin1* 4# at a ratio of 1:1 (termed lenti-si-*gremlin1*) or lenti-si-NC at a multiplicity of infection (MOI) of 40 with 5 μg/mL polybrene. Cells were collected at 72 h post-infection for western blot assay.

In miRNA experiments, the HSC-T6 cell line was transfected with miRNA mimics using TuboFect Transfection Reagent. Fluorescent labeling of miRNA mimic transfectants was observed under a fluorescence microscope (Nikon, Tokyo, Japan) at different time-points to estimate transfection efficiency. An optimal concentration of 50 nM miRNA mimics was employed for all transfection experiments. After culturing for 24 or 48 h, cells were collected for luciferase and western blot assays, respectively.

### Generation of gremlin1 stable expressing cell line

HSC-T6 cells were transfected with pcDNA3.1-Gremlin1, and then selected using 400 μg/ml of G418 (invitrogen, Thermo Scientific, USA) after 24 h transfection, the emerged single cell colony was collected and transferred to fresh dish to foster for several days. For confirming of gremlin1 expression, the partial of selected cells were harvested at appropriate time and lyzed to perform western bolting assay to detect the expression of gremlin1 using anti-gremlin1 antibody. The clone of the HSC-T6 cells with high level gremlin1 expression was chosen and regarded as Gremlin1-HSC cell for use.

### RNA isolation, reverse transcription and PCR

HSC-T6 cells were homogenized on ice with 1 mL and 800 μl TRIzol reagent (Invitrogen, Thermo Scientific, USA), respectively. Total RNAs were extracted and reverse-transcribed to cDNA according to the manufacturer's instructions (Fermentas, Thermo Scientific, USA), followed by quantification by measuring the ratio of absorbance at 260 nm and 280 nm (A_260_/A_280_= 1.8–2.0) with the Multiskan spectrum instrument (Thermo Scientific, USA). All primers for polymerase chain reaction (PCR) were synthesized by Sangon Biotech (Shanghai, China).

### Western blot analysis

Cells were harvested and lysed in lysis buffer (25 mmol/L Tris-HCl pH 7.5, 2.5 mmol/L ethylene diamine tetraacetic acid (EDTA), 137 mmol/L NaCl, 2.7 mmol/L KCl, 1% sodium deoxycholic acid, 0.1% SDS, 1% Triton X-100, and 2 mmol/L Phenylmethanesulfonyl fluoride (PMSF) and a protease inhibitor cocktail for 30 min at 4°C (Sigma-Aldrich Corp. USA). The supernatant fractions were transferred to a fresh tube after centrifugation at 20,238xg for 30 min at 4°C using a Centrifuge 5424 R (Eppendorf, Hamburg, Germany). Protein concentrations were measured using a BCA Protein Assay kit (Thermo Scientific, USA). An equal amount of protein from each sample was separated via sodium dodecyl sulfate-polyacrylamide gel electrophoresis (SDS-PAGE) and transferred to polyvinylidene difluoride membrane incubated with different primary antibodies for 12 h at 4°C. After being washing with TBST buffer (20 nM Tris-HCl pH 7.5, 150 mmol/L NaCl, 0.05% Tween-20), sections were incubated with secondary antibody for 45 min at room temperature. Membranes were subsequently washed with TBST. Chemiluminescence on the membrane was detected using the ChemiQ 4800mini imaging system (Ouxiang, Shanghai, China). Densitometric analyses of band intensities were performed using ImageJ software (version 1.38×; National Institutes of Health, USA). Primary and secondary antibodies as well as dilution ratios for western blot assays are listed in Table 3.

**Table 3:** Primary antibodies and secondary antibodies for western blotting assay

primary antibody	dilution ratio	company	secondary antibody	dilution ratio	company
Gremlin1	1: 800	Santa Cruz	HRP labeled rabbit anti goat IgG	1:12000	NovoGene
TGF-β1	1: 1000	Santa Cruz	HRP labeled goat anti rabbit IgG	1:8000	NovoGene
Smad2/3	1: 800	Santa Cruz	HRP labeled rabbit anti goat IgG	1:15000	NovoGene
P-Smad2/3	1: 800	Santa Cruz	HRP labeled rabbit anti goat IgG	1:15000	NovoGene
Smad1	1: 800	Santa Cruz	HRP labeled goat anti mouse IgG	1:6000	NovoGene
P-Smad1	1: 800	Santa Cruz	HRP labeled rabbit anti goat IgG	1:15000	NovoGene
BMP-7	1: 1000	Santa Cruz	HRP labeled rabbit anti goat IgG	1:12000	NovoGene
Collagen Iα1	1: 800	Santa Cruz	HRP labeled rabbit anti goat IgG	1:12000	NovoGene
Collagen Iα2	1: 1000	Santa Cruz	HRP labeled rabbit anti goat IgG	1:12000	NovoGene
α-SMA	1: 1000	Sigma	HRP labeled goat anti mouse IgG	1:6000	NovoGene
β-actin	1: 3000	Sigma	HRP labeled goat anti mouse IgG	1:6000	NovoGene

### Luciferase reporter gene assay

To confirm whether mimic miRNAs bind to *gremlin1* mRNA 3′-UTR, HSC-T6 cells transfected with mimic miRNAs were co-transfected with pMIR-Luc-3′-UTR for 24 h, collected for the luciferase assay by centrifugation at 1000 rpm for 3 min at 4°C, lysed in 1 ×Cell Culture Lysis Reagent (Promega, USA) for 10 min at 4°C, and the supernatant transferred to a fresh tube after centrifugation at 10,000 rpm for 20 min at 4°C. Luciferase assays were performed using the Luciferase Assay System (Promega) according to the manufacturer's instructions. Reactions were examined using a Fluorescence Detector (Brethold, Bad Wildbad, Germany), and the protein concentrations measured using a BCA Protein Assay kit (Thermo Scientific, USA).

### Hepatic fibrosis rat model and histopathology

Approval for animal experiments was obtained from the Animal Ethics Committee of the Animal Laboratory Center of China Three Gorges University (CTGU), prior to the research. All animal experiments were performed in specific pathogen-free facilities at the Animal Laboratory Center of CTGU. Rats received humane care in compliance with the criteria outlined in the “Guide for the Care and Use of Laboratory Animals”. Rats were purchased from the Disease Control Center of the Hubei Province (China), and acclimatized to the appropriate conditions for a week before *in vivo* studies. All the methods were carried out in accordance with the relevant guidelines, including any relevant details.

Healthy male Sprague Dawley (SD) rats (*n* = 24, 180–220 g) were randomly divided into four groups. The rats in groups 1, 2, and 3 (*n* = 6) were intraperitoneally injected with 40% CCl_4_ (0.2 ml/100 g) in corn-oil twice per week for 7 weeks. Control rats in group 4 (*n* = 6) were injected with the same dose of corn-oil. Once the rat liver fibrosis model was established, animals in groups 1 and 4 received 300 μL corn-oil and those in groups 2 and 3 received 300 μL of 10^9^ TU/ml lenti-si-NC or lenti-si-*gremlin1* via tail vein injection each day. All the rats were anesthetized with urethane after 2 weeks of lentivirus injection and sacrificed. The whole livers were collected for histological, immunofluorescence and collagen content analyses.

The liver tissues of the rats were harvested, fixed in 10% neutral buffered formalin for 24 h, processed, and embedded into paraffin blocks. Routine Hematoxylin and Eosin (H&E) staining was performed on 5 μm sections of tissue cut from formalin-fixed, paraffin-embedded (FFPE) blocks. Morphology of liver and proliferation of total collagen were analyzed using H&E and Masson's trichrome stain, respectively. The collagen fiber area was quantified using a multifunctional microscope and image analysis system (Leica, Wetzlar, Germany).

### Immunofluorescence

Formalin-fixed, paraffin-embedded (FFPE) tissue blocks were sectioned at 5 μm, baked for 1.5 h for deparaffinization at 65°C, and rehydrated prior to antigen retrieval using a standard xylene/alcohol protocol. Endogenous peroxidase activity was blocked with 3% hydrogen peroxide in TBST 5 min. An antigen retrieval step in citrate buffer (10 mM, pH 6) for 10 min under low microwave power was conducted. The blocker was drained, and gremlin1 and BMP-7 (Santa Cruz, USA) primary antibodies were applied at a concentration of 1 μg/mL. Control sections were incubated with the appropriate IgG negative control. Slides were incubated at 37°C for 1 h, followed by overnight incubation at 4°C with primary antibodies at 1:50 dilution, and subsequently for 1 h at 37°C with FITC-labeled rabbit anti-goat IgG (H+L) (NovoGene Biotech, Wuhan, China). Sections were contrasted and imaged under a TS100 fluorescence microscope (Nikon, Tokyo, Japan).

### Collagen content assay

Collagen content was determined using the total collagen kit (Quickzyme, Leiden, Netherlands) based on the detection of hydroxyproline. Following the manufacturer's protocol, liver tissue samples were hydrolyzed in 6 M HCl at 95°C for 20 h, ultimately resulting in a chromogen with an absorbance maximum at 570 nm.

### Statistical analysis

Data are presented as mean ± standard deviation (SD) of several experiments. Difference between two groups was analyzed by a two-tailed Student's *t*-test, and difference between three or more groups was analyzed by one-way ANOVA multiple comparisons. *P* < 0.05 was considered statistically significant.

Authors are grateful to Professor Hong-Bing Zhang at Peking Union Medical College and Chinese Academy of Medical Sciences for critical reading of the manuscript and Professor Yan-Jie Lv at Harbin Medical University for gifting the plasmid pMIR-REPORTTM Luciferase. We also thank Shan-Bing Yin for English language polishing.

## SUPPLEMENTARY MATERIALS FIGURE


