# Supplementary material for: Suppression of hepatic stellate cell activation through downregulation of gremlin1 expression by the miR-23b/27b cluster
Source: Oncotarget. 2016 Nov 15;7(52):86198–210. doi: 10.18632/oncotarget.13365 (PMC5349907; doi:10.18632/oncotarget.13365)
Supplement: Supplementary file 1 [file oncotarget-07-86198-s001.pdf]

Suppression of hepatic stellate cell activation through downregulation of gremlin1 expression by the miR-23b/27b cluster

Supplementary Materials

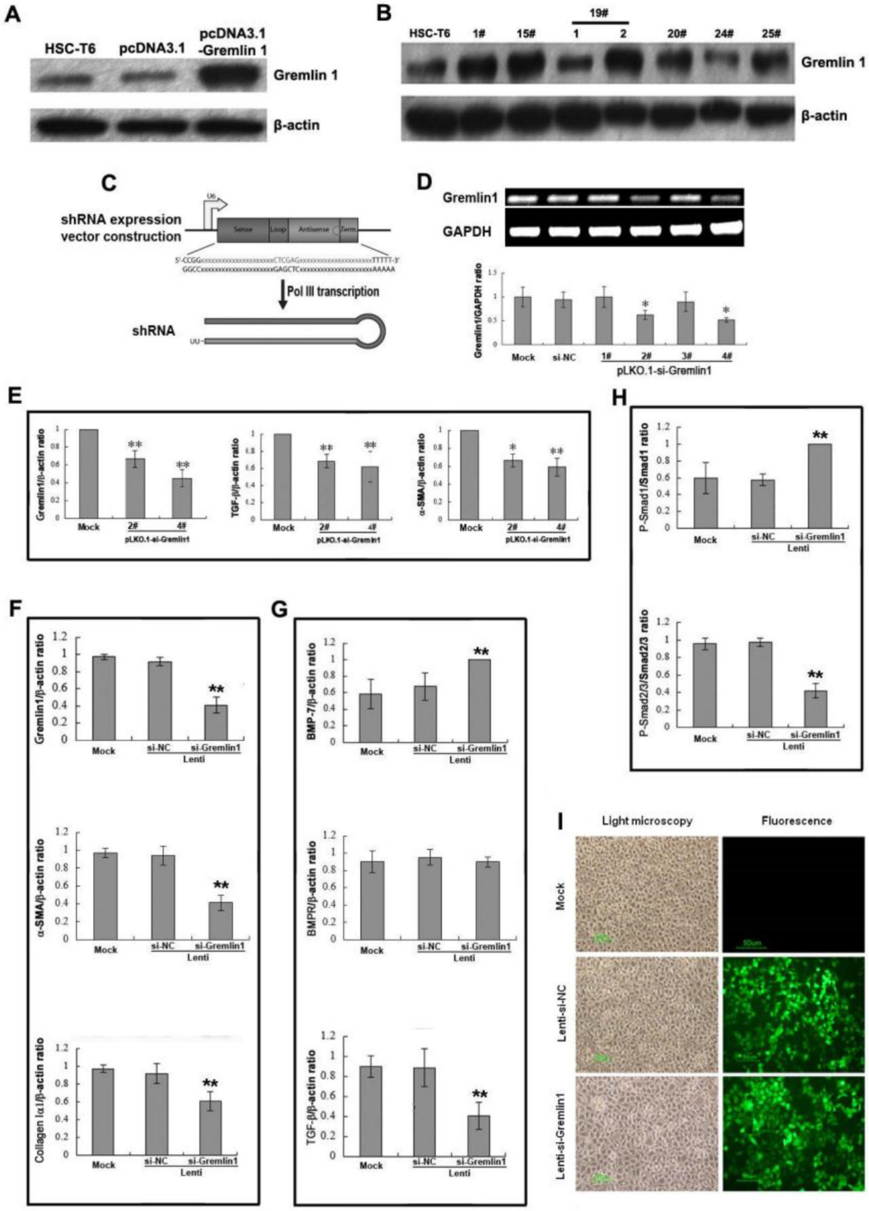

Supplementary Figure S1: Western blots confirming pcDNA3.1-Gremlin1 expression, RT-PCR screening of siRNA efficiency, and quantitative data for western blot. (**A**) Detection of transient expression of gremlin1 in HSC-T6 cells using western blot. (**B**) Generation of a gremlin1 stable expression cell line by transfecting HSC-T6 cells with pcDNA3.1-Gremlin1, followed by G418 selection. Several clones, including clones 1, 15 and 19-2, stably expressed gremlin1 at a higher level than others, as determined from western blots. Clone 19-2 (termed Gremlin1-HSC) was selected for further experiments. (**C**) Illustration of gremlin1-specific shRNA-expressing vector pLKO.1-si-Gremlin1 construction. (**D**) HSC-T6 cells were transfected with gremlin1-specific shRNA-expressing vectors, pLKO.1-si-Gremlin1 1#, 2# 3# or 4#, and total RNA isolated from cells cultured in each well used for semi-quantitative RT-PCR. 2# and 4# shRNA downregulated gremlin1 expression, compared with non-transfected or scrambled shRNA-transfected cells. (**E–H**) Quantitative data for western blots of the indicated proteins from HSC-T6 transfected or infected with gremlin1 shRNA expressing plasmid or virus, respectively. (**I**) Transfection efficiency of Lenti-si-Gremlin1. HSC-T6 cells were transfected with Lenti-si-Gremlin1 and Lenti-si-NC. Overall, > 90% cells were observed under a fluorescence microscope.
